# A *Modern* Approach to Disinfection, as *Old* as the Evolution of Vertebrates

**DOI:** 10.3390/healthcare2040516

**Published:** 2014-12-19

**Authors:** Franco Migliarina, Sergio Ferro

**Affiliations:** 1 Administrative Services Business, ASL AT, via Conte Verde 125, 14100 Asti, Italy; E-Mail: fmigliarina@asl.at.it; 2 Department of Chemical and Pharmaceutical Sciences, University of Ferrara, via Fossato di Mortara, 17-27, 44121 Ferrara, Italy

**Keywords:** hypochlorous acid, water disinfection, *Legionella*, electrochemical synthesis, anolyte

## Abstract

The immune system of vertebrates “naturally” produces hypochlorous acid (HOCl) to fight against bacteria and pathogens. A patented electrochemical technology mirrors the above defense system, allowing the synthesis of HOCl solutions through the electrolysis of water enriched in salts, at the level of a few grams per liter. The system allows for the careful control of the pH of produced solutions, with consequent optimization of their activity. Once the HOCl is introduced into the water system; it is able to remove the biofilm from pipe network; significantly decreasing the level of *Legionella* colonization; within 8–10 weeks from the beginning of the disinfection approach. The technology has been applied in a variety of healthcare facilities, both in Italy and in neighboring European countries. In the present paper, two successful case studies are briefly presented: Data were obtained from experiences in two different healthcare facilities, one in Italy and the other in Germany. Destruction of biofilm was indirectly testified by an increase of total organic carbon content of water; as a consequence, and because of the dosing of the disinfecting agent, some μg/L of total halomethanes were also formed. However, both compositional features were only observed during the initial stages of the disinfection treatment.

## 1. Introduction

Different approaches are currently being used to fight against opportunistic pathogens present in aquatic environments, such as *Legionella*. Since each method has some pros and, unfortunately, some cons, a universally applicable solution has not been identified yet. The approach described in this study was inspired by observing how the immune system of vertebrates works to defend the body from any form of chemical, traumatic or infectious insult to its integrity (see e.g., [1]). Nonspecific immunity is substantially based on the activity of neutrophils and macrophages leukocytes, which have the fundamental task to capture and destroy foreign substances, through phagocytosis. Of great importance is the action of myeloperoxidase (MPO), an enzyme that produces hypochlorous acid (HOCl) from hydrogen peroxide (H_2_O_2_) and chloride anion (Cl^−^), during the neutrophils’ respiratory burst [2,3,4].

Hypochlorous acid possesses a particularly effective cytotoxic activity [5,6,7]. Unfortunately, in contrast to other forms of so-called *active chlorine* (*i.e.*, hypochlorite and gaseous chlorine), the HOCl molecule is rather unstable, and cannot be stored for long periods and used at request. To produce it, three different methods can be utilized [4]: Hydrolysis of chlorine, electrolysis of chloride-containing solutions, and acidification of hypochlorite. The first and third of the methods have drawbacks and dangers, mainly related with the use or the possible release of gaseous chlorine. As a result, the easiest and safest synthetic path is electrochemical production, through electrolysis of a brine solution. Various approaches exist, which are all based on electrochemical cells, provided with or without a separator to avoid reaction or decomposition of products synthesized at the anodes upon contact with the cathodes. Only a few of them allow the synthesis of a biocide solution under well-controlled and reproducible conditions, and the number of useful devices is further reduced when the constraint of a product with a neutral pH is introduced. The latter is important, as it is linked with the reactivity of the active ingredient [8,9,10,11] and of related chemical equilibria (*i.e.*, conversion of the active chlorine to chlorite and chlorate). In addition, a neutral pH is safer for both the user and the target applications (e.g., minimization of corrosion problems).

**Figure 1:**
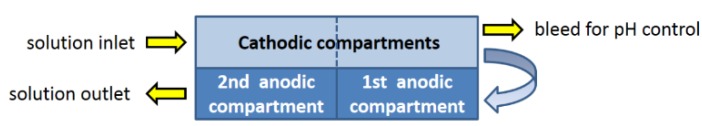
Schematic representation of the electrochemical reactor with four chambers.

**The Ecas4 Approach.** To meet with the above requirements, an Italian company (Ecas srl) has recently patented a technology that relies upon a reactor with four chambers [12], as sketched in Figure 1. As described in the patent application, the incoming solution (having a flow rate comprised between 40 and 160 L/h, depending on reactor size) is distributed between two cathode compartments, and is thus subjected to a single cathodic treatment (cathodes made of titanium are used [13]). Subsequently, the solution is passed through two anodic treatments in series (typically, the anodes are titanium supports covered with an Iridium-based mixed oxide coating; ruthenium is accurately avoided, being for example not allowed by Italian legislation [13]).

By feeding the electrochemical reactor with 24V DC, and operating with a dilute brine solution that contains 0.4%–0.5% of NaCl, about 50 A of current are obtained (electrode current density around 250 A/m^2^), which allow the synthesis of 350–400 mg/L of *active chlorine*. With reference to the anodic treatments, based on a flow rate of 100 L/h and considering that about 10% of the incoming flow is discarded to control the pH, residence times close to 0.35 s can be estimated.

The Ecas approach may be considered as an optimization of the technology for electrochemical activation of water proposed by the Russian school [14,15], allowing the production of the so-called anolyte: A solution containing hypochlorous acid. A given pH is rigorously maintained at the desired value by the apparatus through the controlled discharge of part of the catholyte. The suitable bleeding of the latter is accomplished by means of an electric valve, whose functioning is driven by a pH sensor placed on the anolyte output. A conductivity sensor controls the salt content in the diluted brine, and another device measures the oxidation-reduction potential of the anolyte. When a parameter is outside the designated range, the synthesized product is discarded, while automatic regulators try to correct the anomaly. As a result, an anolyte with constant and reproducible characteristics is synthesized, irrespectively from variations in water pressure or other inconveniences (e.g., wear of the catalytic electrode coating).

The benefits deriving from the use of hypochlorous acid (active chlorine at pH ≤ 7.5), compared to those of using hypochlorite (active chlorine at pH > 7.5) can be summarized as follows:

1. Higher disinfectant efficacy of HOCl, with respect to ClO^−^ (about two orders of magnitude, as demonstrated also experimentally; see e.g., [16]); accordingly, the use of HOCl makes it possible to obtain results comparable to those attainable with the use of hypochlorite, but using lower concentrations.
2. HOCl apparently behaves like a source of hydroxyl radicals, rather than as a chlorine-containing oxidizing agent, thus minimizing the risk of formation of undesired byproducts [17].
3. A neutral solution does not alter the pH characteristics of the treated liquid (often a potable water), and provides greater assurance concerning possible problems of corrosion for metal piping.

With a second patent application [18], the technology has been further improved, borrowing the zero-gap principle, *i.e.*, with electrode in direct contact with the separating membrane, from the fuel cell and chlor-alkali industries (see e.g., [19]). This allows to reduce the salinity of the diluted brine (with benefits in terms of stability of the anolyte and minimization of non-active chemicals), while reducing the possible heating due to ohmic drop (in general, heat is deleterious, both from a chemical point of view as for the stability of the electrodes and of the membrane).

Considering Italy and a few neighboring European countries (Spain, Germany, Slovenia), the effectiveness of the above-discussed anolyte, for the control and possible eradication of *Legionella* in healthcare facilities, has been extensively verified. In most cases, the same healthcare facilities have carried out comparative tests, in order to attest the effectiveness of the anolyte-based disinfecting system, in comparison to other approaches (systems based on monochloramines, hydrogen peroxide and silver, chlorine dioxide, UV disinfection, thermal shocks). Unfortunately, healthcare facilities are usually not prone to share all obtained results, as this would mean admitting problems of contamination within the water network of the structure. Thus, independent tests have been also carried out by different university groups (in Italy, Germany and Slovenia) in order to investigate on the various aspects of the application. In relation to the applicability to water supply systems, attention has been specifically addressed to rule out possible problems due to corrosiveness of the anolyte, and to the verification of its biocide effectiveness against major pathogens (*i.e.*, *Pseudomonas aeruginosa*, *Staphylococcus aureus*, *Escherichia coli*, *Aspergillus niger*, *Candida albicans*). Referring also to the work by other groups, the list of pathogens against which anolytes have been tested so far is impressive, as underlined in a recent review by Reynolds and coworkers [20].

The present study aims at reporting and briefly discussing some of the data obtained from experiences in two healthcare facilities, one in Italy and the other in Germany.

## 2. Methods

The investigations were carried out at the University Hospital of Dresden (Germany) and at the Cardinal Massaia Hospital, Asti (Italy).

*University Hospital of Dresden, Dresden, Germany.* Due to the positioning of the buildings in a pavilion system, the Dresden University Hospital has used several different hot water systems decontamination methods. Besides the electrolytic process presently under discussion, thermal disinfection, a combination of UV with ultrasound or the filtration of drinking water at the point of use have all been used. Despite several thermal decontaminations, colony counts of *Legionella pneumophila* in the hot water system of the department of neurology were repeatedly over 10,000 colony-forming units (CFU)/L. Isolates were determined to belong to several serogroups, including serogroup 1 that is associated with a higher virulence. A two-story building of 15 years with 45 beds was selected to assess the performance of the Ecas technology.

*Cardinal Massaia Hospital, Asti, Italy.* The Asti hospital is organized in six departments, for a total of 471 beds for inpatient admissions and 51 places for day-hospital. In addition, a surgical unit with 11 rooms, a block of Day Surgery and an obstetric room are located on the −1, 2nd and 3rd floor, respectively. The total area of the Asti hospital amounts to 125,000 m^2^; the daily average attendance is about 450 patients, and the employment index is equal to 90%. Nosocomial infections are prevented by using control measures against *Legionella* proliferation in the hot water distribution system. The plant is divided into several sub-stations: Each one consists of a heat exchanger, fed by water at 90 °C coming from the main heating plant, 11 vertical tanks and 1 horizontal tank in galvanized steel, internally coated with Teflon. The distribution plant consists of a recirculation loop, in order to keep an optimal temperature in the vicinity of the different peripheral points. The cold water pertaining to the hot water system network is softened at 4 °f. In general, a complete elimination of bacteria is difficult to achieve with any disinfection approach. In this study, the efficacy of two continuous dosing methods for the eradication of *Legionella* from hospital water supplies has been evaluated and compared. Both approaches require the continuous dosing of a biocide into the hot water system: Method 1 involves the use of an electrochemically activated water (the Ecas anolyte), containing hypochlorous acid at neutral pH, while method 2 relies upon a solution of hydrogen peroxide and silver (Cillit Allsil Super 25 Ag). It is worth mentioning that the latter approach is not always applicable, being for example not permitted by German legislation for continuous treatment of drinking water [21]. The two continuous disinfection systems were installed in the Asti hospital in two distinct water supplies.

In both cases (Dresden and Asti hospitals), the composition of the just-prepared anolyte was as follows: Hypochlorous acid 325 mg/L; pH 7.0 ± 0.1; oxidation-reduction potential (ORP) 850–900 mV; chloride content less than 0.5%.

### 2.1. Sample Collection

Between November 2009 and October 2010, hot water samples were collected from different points of use (showers, sinks) within building 62, Dept. of Neurology, at the Dresden Hospital; the apparatus for the anolyte synthesis was installed and started operating on August 11, 2010.

Concerning the Cardinal Massaia Hospital (where the investigation was launched in late 2008), two samplings from seven different points, within each water system, were performed before the installation of the disinfection plants, and then repeated after three weeks from the installation of the disinfection systems. Subsequently, eight samplings were periodically performed for 5 months (every 2 weeks, during the first 3 months, and once a month for the subsequent 2 months). In total, 70 samples were analyzed for each system.

In all cases, water sampling was performed by means of large containers (>5 liters, collected by leaving an air space in the bottle), adding an excess of sodium thiosulfate (~100 mg, used as inactivating agent) to specimens that contain a residual biocide content. Samples were collected both before and after flushing operations (since stagnant water favors *Legionella* growth, different results are expected).

Cultures were performed following standard quantitative protocols (detection limit: 20 CFU/L) [22,23]. Samples (5 liters each) were concentrated by filtration on a 0.2 μm pore-diameter polycarbonate membrane (Millipore, Ireland); then, the suspensions were re-suspended in 5 mL of water and shaken vigorously. Heat treatment for selective inhibition of non-*Legionella* bacteria were performed as described in [23]. Centrifuged and re-suspended suspensions (1 mL) were sub-cultured on BCYE, BMPA and MWY agar [24] (three replicates, dispensing 0.1 mL each), incubated at 37 ± 1 °C for 15 days, to allow *Legionella* colonies counting and typing. Results were reported as total counts of *Legionella*.

The residual biocide content in water samples was quantified during sampling by means of the standard N,N-diethyl-1,4-phenylenediamine colorimetric method [25]. Redox and pH were measured by means of a multiparametric probe.

### 2.2. Biocide Dosing

Once produced, the anolyte is injected in the water network of the structure by means of a diaphragm pump, which must be sized depending on the amount of water to be treated. The concentration of the active ingredient in the anolyte was always between 300 and 400 mg/L (rather low when compared to other forms of chlorine-based disinfectants). The pump is capable of injecting up to a distance of 120 meters and can operate in volumetric or proportional mode. On-line measurement of anolyte concentration takes place by means of a potentiostatic probe, which sends the result to a residual chlorine analyzer, connected to the injecting pump. Two different probes are typically used: One in relatively close proximity and the other located far from the injection point; in this way, the biocide can be suitably dosed, without exceeding the maximum residual disinfectant limits set by the legislation in force.

## 3. Results and Discussion

### 3.1. Decontamination of Hot Water System — Dresden University Hospital (Germany)

At the beginning of the monitoring program (see Table 1), analyses showed a systemic colonization of the hot water network of the department of Neurology: Colony counts of *Legionella pneumophila* were repeatedly over 10,000 CFU/L. Within a week from the installation of the HOCl generating unit, a significant reduction in colony counts of *L. pneumophila* at water outlets was achieved. Colony counts increased slightly afterwards, plausibly because of the destruction of biofilm. However, the contamination completely disappeared after about 8 weeks. No *Legionella* was detected 6 months later during the manufacturer’s recommended inspection. Technical problems such as corrosion or leaching of the piping were not reported. During the first 8 weeks, chemical analyses have determined that the chlorine content in tap water did not exceed 0.6 mg/L. Actually, the German regulation allows a chlorine content in drinking water up to 1.2 mg/L at the injection point, and between 0.1 and 0.3 mg/L at the points of use; exceptionally, the chlorine concentration can be raised up to 0.6 mg/L [21,26]. Patients and staff tolerated the temporary slightly higher chlorine smell.

**Table 1:** Selection of data on *Legionella* detection (values expressed in colony-forming units (CFU)/L); Department of Neurology, Dresden University Hospital. Asterisks refer to withdrawals performed with the electrochemical technology implemented in the water system (the electrochemical technology was put into operation on 11.08).

**Sample**	**Notes**	**02.11**	**15.11**	**01.12**	**09.02**	**15.06**	**02.08**	*16.08	*23.08
shower in room 124	before flushing	2000	1000			100,000	10,000	0	1,000
	after flushing	6000	2000			33,000	3000	0	0
Shower in room 134	before flushing	1000	1000	2000	0	3000	0	0	0
	after flushing	8000	11,000	5000	7000	11,000	5000	0	4
Sink in room 134	before flushing		12,000	1000	0	40	0	0	0
	after flushing		14,000	6000	15,000	3000	2000	0	1000
Sink in room 61	before flushing	1000		10,000	3000	7000	1000	100	2,000
	after flushing	16,000		3000	6000	7000	2000	20	2,000
**Sample**	**Notes**	*30.08	*06.09	*13.09	*20.09	*27.09	*11.10	*18.10	*25.10
Shower in room 124	before flushing	3000	0	20	0	1000	0	0	0
	after flushing	280	0	0	0	4000	0	0	0
Shower in room 134	before flushing	1000	0	0	0	0	0	0	0
	after flushing	1000	0	0	0	1000	0	0	0
Sink in room 134	before flushing	4000	0	0	0	2000	0	0	0
	after flushing	4000	0	0	0	1000	0	0	0
Sink in room 61	before flushing	28,000	14,000	70	0	2000	0	0	0
	after flushing	15,000	1000	20	0	4000	0	0	0

Obtained results (Table 1) demonstrate an immediate reduction and eventual eradication of microbial contamination by *Legionella* in the water system, after about two months of operation.

### 3.2. Evaluation of Two Disinfection Systems for Legionella Eradication — Cardinal Massaia Hospital, Asti (Italy)

Pre-treatment samples from water supply 1 showed *Legionella* contamination of 60–180 CFU/L in the hot water tank, and of 300–16,000 CFU/L in distal points. After starting the anolyte-based disinfection system (method 1: Dosing of *active chlorine* was between 1.2 and 0.3 mg/L, mean 0.6 mg/L), all samples showed a clear reduction in colony counts (≤100 CFU/L). Samples from water supply 2 showed initial contaminations in the range 180–24,000 CFU/L. After starting the disinfection, by dosing 2 mg/L of hydrogen peroxide (method 2), the contamination reduced to 20–15,000 CFU/L. During the observation period, the product was dosed at variable concentrations and only in the second-last sampling, in the presence of the highest dose of biocide (>25 mg/L), the culture counts were negative. However, the contamination appeared again in the last sampling (H_2_O_2_ at 2 mg/L), with values up to 600 CFU/L.

**Figure 2:**
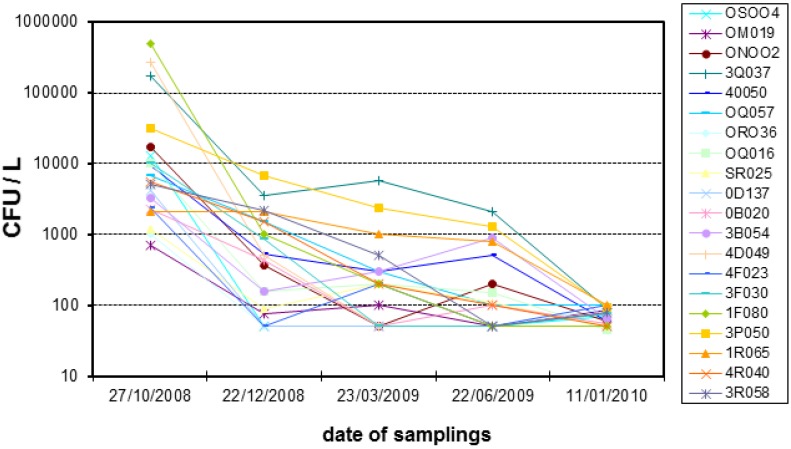
Trend of *Legionella* colony counts, as a function of time, in the first months of operation of the anolyte-based disinfection system (method 1). Cardinal Massaia Hospital, Asti. From 2010 onwards, the contamination remained constantly below 100 CFU/L.

Method 1 proved to be effective in eradicating *Legionella* from the hospital water supply, with *active chlorine* concentration > 0.2 mg/L (the Italian legislation suggests a level of 0.2–0.3 mg/L at the point of use [27]). In contrast, method 2 was not efficient, at least at the biocide concentration proposed by the manufacturer. After the six-months research at one of the twelve hot-water circuits of the large Hospital of Asti, the medical management decided to install the anolyte-producing unit on all hot-water systems and units were installed in all buildings in October-November 2009. Results shown in Figure 2 refer to the first months of operation of the system (the chart legend reports the code of the rooms from where the samples were taken). From 2010 onwards, trends are actually straight lines connecting colony count data comprised between 50 and 100 CFU/L. Occasional rises (never exceeding 300 CFU/L) coincided with maintenance or extended periods of non-utilization [28].

In all cases, the quality of water was investigated before and after the implementation of the anolyte-based system, by analyzing a number of common parameters [29,30]. In particular, the trend of total organic carbon (TOC) and the formation of halogenated byproducts (total halomethanes, THMs, and chloroform) can be reliably related to the functioning of the system, as shown in Table 2. Looking at the data, it is evident that the TOC initially increased, as a result of the implementation of the system in the water network, but subsequently decreased, thus suggesting that the biofilm was initially attacked and then destroyed. Since organic substances represent a suitable substrate for chlorination reactions (which are plausibly due to the hypochlorite fraction of the dosed biocide), some THMs are formed during biofilm destruction. Since their amount depends on both the biocide dosed and on TOC, it is expected to decrease once the organic matter is eliminated.

**Table 2:** Trend of water parameters, after the implementation of the disinfecting system, in the first months of operation.

Water Sample	TOC (mg/L)	Residual Free Chlorine (mg/L) *	CFU/mL	Total THMs (μg/L)	Chloroform (μg/L)
Before	1.0 ± 0.3	N.A.	200 ± 30	0.5	<0.1
After 2 months	1.1 ± 0.2	0.3 ± 0.1	6 ± 5	3.4	<0.1
After 4 months	2.9 ± 0.6	0.2 ± 0.1	12 ± 7	4.6	<0.1
After 6 months	1.7 ± 0.3	0.3 ± 0.1	2 ± 1	4.1	<0.1

* The dosing of the biocide (up to 1.2 ppm at the point of injection) was adjusted to obtain the residual concentration reported.

The technology is currently applied in many different healthcare facilities, in Italy, Spain, Germany and Slovenia. Test results required by the Australian Water Quality Centre (NATA approved laboratory) confirmed the effectiveness of anolyte against targeted organisms (*Legionella pneumophila*, *Pseudomonas aeruginosa* and *Escherichia coli*); as a result, negotiations are presently in progress with a healthcare facility in Australia to install the first machine.

Since also microorganisms belong to the class of “organic substrates”, the anolyte-based approach may be considered as a form of indirect oxidation of organic substances [31,32]. The alternative direct oxidation [33,34,35,36] undoubtedly represents an interesting approach, maybe also more efficient (the inactivation of microorganisms does not rely only upon the synthesis of a biocide, being also consequence of electric field effects [37] and sudden variations of pH) but the size of the reactor required to treat the entire water flow makes this method often impracticable.

## 4. Conclusions

The two case studies have shown that implementation of an *in situ* electrochemical technology for synthesis and dosing of hypochlorous acid eradicated *Legionella* contamination from two large hospital water systems.

Although the considered healthcare facilities did not provide us with all available data, the evidences are unquestionably in favor of the effectiveness of the proposed anolyte-based approach. In the two considered case studies, the *Legionella* colony counts decreased significantly within a few weeks from the beginning of the disinfection treatment. The total organic carbon content of the treated water showed to increase, as plausible consequence of the destruction of biofilm. As a temporary effect, also the level of THMs increased, anyway remaining well below the legislated limit (e.g., 30 μg/L in Italy). The technology demonstrates great potential for applicability in a variety of settings, with no negative consequences to infrastructure or potable water aesthetics. More information on implementation and performance of this system is clearly warranted.

## References

[1] Frank S.A. (2002). Immunology and Evolution of Infectious Disease.

[2] Klebanoff S.J. (2005). Myeloperoxidase: Friend and foe. J. Leukocyte Biol..

[3] Hoffstein S.T., Gennaro D.E., Manzi R.M. (1985). Neutrophils may directly synthesize both H_2_O_2_ and O_2_^−^ since surface stimuli induce their release in stimulus-specific ratios. Inflammation.

[4] Wang L., Bassiri M., Najafi R., Najafi K., Yang J., Khosrovi B., Hwong W., Barati E., Belisle B., Celeri C. (2007). Hypochlorous acid as a potential wound care agent. Part I. Stabilized hypochlorous acid: A component of the inorganic armamentarium of innate immunity. J. Burns Wounds.

[5] Hampton M.B., Kettle A.J., Winterbourn C.C. (1998). Inside the neutrophil phagosome: Oxidants, myeloperoxidase, and bacterial killing. Blood.

[6] Klebanoff S.J. (1999). Mieloperoxidase. Proc. Assoc. Am. Physicians.

[7] Winterbourn C.C. (2002). Biological reactivity and biomarkers of the neutrophil oxidant, hypochlorous acid. Toxicology.

[8] Len S.V., Hung Y.C., Erickson M., Kim C. (2000). Ultraviolet spectrophotometric characterization and bactericidal properties of electrolyzed oxidizing water as influenced by amperage and pH. J. Food Prot..

[9] Abadias M., Usall J., Oliveira M., Alegre I., Viñas I. (2008). Efficacy of neutral electrolyzed water (NEW) for reducing microbial contamination on minimally processed vegetables. Int. J. Food Microbiol..

[10] Xiong K., Liu H.-J., Liu R., Li L.-T. (2010). Differences in fungicidal efficiency against *Aspergillus flavus* for neutralized and acidic electrolyzed oxidizing waters. Int. J. Food Microbiol..

[11] Neodo S., Rosestolato D., Ferro S., de Battisti A. (2012). On the electrolysis of dilute chloride solutions: Influence of the electrode material on Faradaic efficiency for active chlorine, chlorate and perchlorate. Electrochim. Acta.

[12] Bohnstedt R., Surbeck U., Bartsch R. (2009). Membrane electrolytic reactors system with four chambers. European Patent n..

[B13-healthcare-02-00516] 13.Regolamento concernente i materiali e gli oggetti che possono essere utilizzati negli impianti fissi di captazione, trattamento, adduzione e distribuzione delle acque destinate al consumo umano. D. Min. 174, 17.07.2004, G.U. n. 166 (In Italian).

[14] Bakhir V.M. (1985). Regulating physical and chemical properties of technological aqueous solutions by unipolar electrochemical exposure and experience of its practical application. Ph.D. Thesis.

[15] Prilutsky V.I., Bakhir V.M. (1997). Electrochemically Actuating Water: Anomalous Characteristics, Mechanism of Biological Action.

[16] Faust S.D., Aly O.M. (1998). Chemistry of Water Treatment.

[17] Tamburini E., Bernardi T., Castaldelli G., Tumiatti G., Ferro S. (2011). Green electrochemical approach for delignification of wheat straw in second-generation bioethanol production. Energy Environ. Sci..

[18] Quadrelli S., Ferro S. (2010). Electrochemical Reactor. Intern. Pat. Appl. n..

[19] Lee J.S., Quan N.D., Hwang J.M., Lee S.D., Kim H., Lee H., Kim H.S. (2006). Polymer electrolyte membranes for fuel cells. J. Ind. Eng. Chem..

[20] Thorn R.M.S., Lee S.W.H., Robinson G.M., Greenman J., Reynolds D.M. (2012). Electrochemically activated solutions: Evidence for antimicrobial efficacy and applications in healthcare environments. Eur. J. Clin. Microbiol. Infect. Dis..

[21] Liste der Aufbereitungsstoffe und Desinfektionsverfahren, Trinkwasserverordnung 2008, gemäβ § 11—Teil I c. http://www.lra-mue.de/shared/data/pdf/liste_paragraf_11_stand_juni_2008.pdf.

[B22-healthcare-02-00516] 22.Italian guidelines for prevention and control of Legionellosis. 05.05.2000, G.U. n. 103.

[23] International Organization for Standardization (ISO) Water Quality: Detection and Enumeration of Legionella.

[24] Descours G., Cassier P., Forey F., Ginevra C., Etienne J., Lina G., Jarraud S. (2014). Evaluation of BMPA, MWY, GVPC and BCYE media for the isolation of Legionella species from respiratory samples. J. Microbiol. Methods.

[25] International Organization for Standardization (ISO) Colorimetric method using N,N-diethyl-1,4-phenylenediamine, for routine control purposes. Water Quality: Determination of Free Chlorine and Total Chlorine.

[26] Verfahren zur Desinfektion von Trinkwasser mit Chlor und Hypochloriten, Arbeitsblatt W 229. 2008; DVGW technical rule. http://www.beuth.de/de/technische-regel/dvgw-w-229/110879513.

[B27-healthcare-02-00516] 27.Attuazione della direttiva 98/83/CE relativa alla qualità delle acque destinate al consumo umano. D. Lgs. n. 31, 03.03.2001, G.U. n. 52.

[28] Migliarina F. (2014). Personal Communication.

[29] American Public Health Association (APHA), American Water Works Association (AWWA), Water Environment Federation (WEF) (2005). Standard Methods for the Examination of Water and Wastewater.

[30] APAT, IRSA-CNR (2003). Metodi analitici per le Acque.

[31] Martinez-Huitle C.A., Ferro S. (2006). Electrochemical oxidation of organic pollutants for the wastewater treatment: Direct and indirect processes. Chem. Soc. Rev..

[32] Ghernaout D., Naceur M.W., Aouabed A. (2011). On the dependence of chlorine by-products generated species formation of the electrode material and applied charge during electrochemical water treatment. Desalination.

[33] Bonfatti F., Ferro S., Lavezzo F., Malacarne M., Lodi G., de Battisti A. (1999). Electrochemical incineration of glucose as a model organic substrate. Part 1: Role of the electrode material. J. Electrochem. Soc..

[34] Martinez-Huitle C.A., de Battisti A., Ferro S., Reyna S., Cerro-Lopez M., Quiroz M.A. (2008). Removal of the pesticide Methamidophos from aqueous solutions by electrooxidation using Pb/PbO_2_, Ti/SnO_2_ and Si/BDD electrodes. Environ. Sci. Technol..

[35] Ferro S., Martinez-Huitle C.A., de Battisti A. (2010). Electroxidation of oxalic acid at different electrode materials. J. Appl. Electrochem..

[36] Furuta T., Tanaka H., Nishiki Y., Pupunat L., Haenni W., Rychen P. (2004). Legionella inactivation with diamond electrodes. Diamond Relat. Mater..

[37] Chen D.-Q., Huang S.-S., Lu Y.-J. (2006). Efficient transformation of Legionella pneumophila by high-voltage electroporation. Microbiol. Res..

